# Multi-omics pan-cancer study of cuproptosis core gene *FDX1* and its role in kidney renal clear cell carcinoma

**DOI:** 10.3389/fimmu.2022.981764

**Published:** 2022-12-20

**Authors:** Jiahao Xu, Zhengang Hu, Hui Cao, Hao Zhang, Peng Luo, Jian Zhang, Xiaoyan Wang, Quan Cheng, Jingbo Li

**Affiliations:** ^1^ Department of Gastroenterology, The Third Xiangya Hospital, Central South University, Changsha, China; ^2^ Department of Neurosurgery, Xiangya Hospital, Central South University, Changsha, China; ^3^ Brain Hospital of Hunan Province, The Second People's Hospital of Hunan Province, Changsha, China; ^4^ Department of Oncology, Zhujiang Hospital, Southern Medical University, Guangzhou, China; ^5^ National Clinical Research Center for Geriatric Disorders, Xiangya Hospital, Central South University, Hunan, China

**Keywords:** cuproptosis, *FDX1*, pan-cancer, immunology, KIRC, notch

## Abstract

**Background:**

The mechanism of copper-induced cellular death was newly discovered and termed cuproptosis. Inducing cuproptosis in cancer cells is well anticipated for its curative potential in treating tumor diseases. However, ferredoxin 1 (*FDX1*), the core regulatory gene in cuproptosis, is rarely studied, and the regulation of *FDX1* in tumor biology remains obscure. A comprehensive pan-cancer analysis of *FDX1* is needed.

**Methods:**

Thirty-three types of tumors were included with paired normal tissues in The Cancer Genome Atlas (TCGA) and the Genotype-Tissue Expression (GTEx) datasets. The interaction between transcription, protein, phosphorylation, and promoter methylation levels was analyzed. Survival, immune infiltration, single-cell *FDX1* expression, *FDX1*-related tumor mutational burden (TMB), microsatellite instability (MSI), stemness, tumor immune dysfunction and exclusion (TIDE), and immunotherapy-related analyses were performed. *FDX1* protein expression was assessed by kidney renal clear cell carcinoma (KIRC) tissue microarray immunohistochemistry. The function of *FDX1* in KIRC was further explored by experiments in 786-O cell lines *in vitro*.

**Results:**

*FDX1* is highly expressed in 15 tumor types and lowly expressed in 11 tumor types. The corresponding changes in protein expression, phosphorylation, and promoter methylation level of *FDX1* have been described in several tumors. Survival analysis showed that *FDX1* was related to favorable or poor overall survival in eight tumors and progression-free survival in nine tumors. Immune infiltration and single-cell analysis indicated the indispensable role of *FDX1* expression in macrophages and monocytes. Multiple established immunotherapy cohorts suggested that *FDX1* may be a potential predictor of treatment effects for tumor patients. Tissue microarray analysis showed decreased *FDX1* expression in KIRC patients’ tumor tissues. Knockdown of *FDX1* resulted in the downregulation of cuproptosis in kidney renal clear tumor cells. Mechanistically, the *FDX1*-associated gene expression signature in KIRC is related to the enrichment of genes involved in the tricarboxylic acid (TCA) cycle, NOTCH pathway, etc. Several NOTCH pathway genes were differentially expressed in the high- and low-*FDX1* groups in KIRC.

**Conclusion:**

Our analysis showed that the central regulatory gene of cuproptosis, *FDX1*, has differential expression and modification levels in various tumors, which is associated with cellular function, immune modulation, and disease prognosis. Thus, *FDX1*-dependent cuproptosis may serve as a brand-new target in future therapeutic approaches against tumors.

## Introduction

Cuproptosis is a newly identified cellular death program published in *Science* in 2022, sparking worldwide concern (1). According to this research, excessive intracellular copper can bind to lipoylated tricarboxylic acid (TCA) cycle proteins, causing their aberrant oligomerization and leading to destabilization of iron-sulfur (Fe-S) cluster proteins, resulting in proteotoxic stress and eventually cell death. Ferredoxin 1 (*FDX1*), also called adrenodoxin, was identified in 1967 as one component of the electron transport system in adrenal steroid hydroxylase (2). It was found to be the upstream regulator of protein lipoylation, and its deletion would boost the resistance to cuproptosis (1). It encodes a small Fe-S protein that transfers electrons from reduced form of nicotinamide adenine dinucleotide phosphate (NADPH) through ferredoxin reductase to the mitochondrial cytochrome P450 like cytochrome P450 family 11 subfamily A member 1 (CYP11A1) (3). It has been reported that *FDX1* is required for the synthesis of steroid hormones or heme A and Fe-S proteins (4). Investigations on the relationship between *FDX1* expression and diseases showed that downregulation of *FDX1* was probably related to the onset of polycystic ovary syndrome (PCOS) (5), while a certain *FDX1* genotype was a potential risk factor for glomerulonephritis immunoglobulin A (IgA) nephropathy (6). Recently, *FDX1* was examined in lung adenocarcinoma, but its role in tumorigenesis remains unclear (7). Thus, with the pioneering works of cuproptosis and sparse data on *FDX1* especially in tumor studies, we intended to carry out a comprehensive and systematic pan-cancer analysis to excavate its potential role in different tumors.

## Materials and methods

### Data acquisition and expression analysis

Transcriptomics along with clinical data of 33 tumors and normal tissues with 10,535 samples from The Cancer Genome Atlas (TCGA) and 7,862 samples from Genotype-Tissue Expression (GTEx) were downloaded from The University of California Santa Cruz (UCSC) Xena (http://xena.ucsc.edu/) (**Table 1**). All data were normalized through log2(tpm+0.001). The methylation β value matrix of these 33 tumors was also acquired from UCSC Xena. The data were calculated and normalized using the champ package in R statistical software 3.6.3, and six *FDX1* methylation probes located in the promoter region were obtained. E-MTAB 1980 cohort and GSE14378 were used as the *FDX1* survival validation cohorts. Five independent immunotherapy cohorts were included in the *FDX1* immunotherapy analysis: Kim (GSE135222) (8), CheckMate 010 (CM-010; NCT01354431) (9), CheckMate 025 (CM-025; NCT01668784) (10), Nathanson_2017 (11), and Lauss_2017 (12). The Kim cohort recruited 27 advanced non-small cell lung carcinoma (NSCLC) patients who were treated with anti Programmed cell death protein 1 (PD-1) /Programmed Cell Death Ligand 1 (PD-L1). CM-010 was a phase II study of nivolumab, and CM-025 was a randomized phase III trial of nivolumab. Patients enrolled in both CM-010 and CM-025 were pathologically confirmed to have advanced clear cell renal cell carcinoma. Nathanson_2017 contained melanoma patients treated with cytotoxic T-lymphocyte antigen-4 (CTLA-4) blockade. Lauss_2017 had stage IV melanoma patients under adoptive T-cell therapy (ACT). The methylation cohort of Kim was from GSE119144. All RNA sequencing (RNA-seq) data were processed in the same way as TCGA data. Proteomics and phosphorylation omics of *FDX1* were derived from the Clinical Proteomic Tumor Analysis Consortium (CPTAC) (13). The expression in the level of methylation, transcription, protein, and phosphorylation was compared between tumor and normal tissues. p < 0.05 indicated a significant difference.

**Table 1:** Basic information of the 33 tumors and matched normal tissues.

The Cancer Genome Atlas	Detail	Tumor	Normal	Genotype-Tissue Expression	Num
ACC	Adrenocortical carcinoma	77	0	Adrenal gland	128
BLCA	Bladder urothelial carcinoma	407	19	Bladder	9
BRCA	Breast invasive carcinoma	1,099	113	Breast	179
CESC	Cervical squamous cell carcinoma and endocervical adenocarcinoma	306	3	Cervix uteri	10
CHOL	Cholangiocarcinoma	36	9	–	–
COAD	Colon adenocarcinoma	290	41	Colon	308
DLBC	Lymphoid neoplasm diffuse large B-cell lymphoma	47	0	Blood	337
ESCA	Esophageal carcinoma	182	13	–	–
GBM	Glioblastoma multiforme	166	5	Brain	1,033
HNSC	Head and neck squamous cell carcinoma	520	44	–	–
KICH	Kidney chromophobe	66	25	–	–
KIRC	Kidney renal clear cell carcinoma	530	72	–	–
KIRP	Kidney renal papillary cell carcinoma	288	32	–	–
LAML	Acute myeloid leukemia	173	0	Bone marrow	337
LGG	Brain lower-grade glioma	523	0	Brain	1,033
LIHC	Liver hepatocellular carcinoma	371	50	Liver	–
LUAD	Lung adenocarcinoma	515	59	Lung	288
LUSC	Lung squamous cell carcinoma	498	50	Lung	288
MESO	Mesothelioma	87	0	–	–
OV	Ovarian serous cystadenocarcinoma	427	0	Ovary	88
PAAD	Pancreatic adenocarcinoma	179	4	Pancreas	167
PCPG	Pheochromocytoma and Paraganglioma	179	3	–	–
PRAD	Prostate adenocarcinoma	496	52	Prostate	100
READ	Rectum adenocarcinoma	93	10	Colon	308
SARC	Sarcoma	262	2	–	–
SKCM	Skin cutaneous melanoma	468	1	Skin	557
STAD	Stomach adenocarcinoma	414	36	Stomach	175
TGCT	Testicular germ cell tumor	148	0	Testis	165
THCA	Thyroid carcinoma	512	59	Thyroid	279
THYM	Thymoma	119	2	Blood	337
UCEC	Uterine corpus endometrial carcinoma	181	23	Uterus	78
UCS	Uterine carcinosarcoma	57	0	Uterus	78
UVM	Uveal melanoma	79	0	–	–

### Survival analysis

Kaplan–Meier survival curves and univariate Cox proportional hazards models were plotted with the R Survival package. For assessment of the clinical features [overall survival (OS) and progression-free survival (PFS)], both the 50% and the optimal survival cutoff value of *FDX1* expression provided by the algorithm were involved in our models. A comparison of survival curves was performed using log-rank tests, with p-values <0.05 considered significant.

### Immune infiltration

TIMER2.0 (14) (http://timer.cistrome.org/) was used to estimate *FDX1*-related immune infiltration in the 33 tumors. The algorithm included TIMER, tumor immune dysfunction and exclusion (TIDE), CIBERSORT-ABS, quanTIseq, and xCELL. Correlations between *FDX1* and immune cells that were consistent among two or three algorithms were regarded as meaningful, and some of them were selected to draw a correlation plot.

### The immune landscape of *FDX1* in tumors

A list of 60 types of immune checkpoint genes (24 inhibitory, 36 stimulatory) (15) was selected. Pearson’s or Spearman’s correlation analysis was applied to determine the expression correlation between *FDX1* and the 60 genes in the 33 tumors.

### Tumor immune single-cell *FDX1* expression

Single-cell gene expression data across all available cell types were downloaded from the Tumor Immune Single-cell Hub (TISCH) (http://tisch.comp-genomics.org/home/), a single-cell RNA sequencing (scRNA-seq) database. Two cell type annotations including major lineage and malignancy were used to present the specific expression of *FDX1*. Single-cell profile of GBM_GSE131928_10X_Adults and KIRC_GSE171306, and the visualization of FDX1 expression were performed by R package Seurat. About 10580 and 11828 high-quality cells were obtained in KIRC and GBM studies separately.

### Tumor mutational burden, microsatellite instability, and stemness analysis

The TMB function from the R package maftools was used. TCGA microsatellite instability (MSI) algorithm was based on a previous study (16). The one-class logistic regression (OCLR) algorithm was applied to calculate the stemness index based on gene expression profiles of TCGA (17).

### Tumor immune dysfunction and exclusion algorithm and PD-1/PD-L1 blockade cohort

TIDE uses a set of gene expression markers to evaluate the immune escape ability of tumor cells. Higher TIDE scores mean a poorer immune checkpoint blockade (ICB) therapy response (18). The TIDE score of each patient was retrieved from the TIDE website (http://tide.dfci.harvard.edu).

### Gene set enrichment analysis in KIRC and other cancer types

Differentially expressed genes (DEGs) were obtained through the Limma package by calculating the differential genes in the high and low *FDX1* expression groups (50% cutoff) and taking the logFC absolute value >0.5, p < 0.05 as the differential locus.

Then, genes were ranked by log2FC and imported into gene set enrichment analysis (GSEA). Then, GSEA was performed using the R package clusterProfiler v3.14.3. GSEA gene sets included HALLMARK REACTOME, Gene Ontology (GO), and Kyoto Encyclopedia of Genes and Genomes (KEGG). Gene sets in which | NES | > 1, p-value < 0.05 and q value < 0.25 were considered as significant.

### Tissue microarray and immunohistochemistry

We obtained the kidney renal clear cell carcinoma tissue microarray from the Outdo Biotech company and the ethics was approved. The *FDX1* antibody (12592-1-AP) was bought from Proteintech (www.ptglab.com). The immunohistochemistry (IHC) experiment was done as follows: 1) Bake slices at 60°C for 30 min, routinely dewaxed and hydrated; 2) Antigen retrieval: Recover antigen with 0.01 M citrate buffer (pH 6.0) under high pressure for 2 min, cool to room temperature, and wash with phosphate buffered saline (PBS) for 5 min × three times; 3) Block endogenous peroxidase with 3% H_2_O_2_-methanol, wash at room temperature for 10 min, and wash with PBS for 5 min × three times; 4) Add normal non-immune animal serum dropwise at room temperature for 10 min; 5) Remove the serum, add the primary antibody dropwise, and refrigerate at 4°C overnight; 6) Wash with 0.1% Tween-20 PBS for 5 min × three times; 7) Add biotin-labeled goat anti-mouse/rabbit IgG dropwise and incubate at room temperature for 10 min; 8) Wash with 0.1% Tween-20 PBS for 5 min × three times; 9) Add streptavidin-peroxidase dropwise and incubate at room temperature for 10 min; 10) Color with Diaminobenzidine (DAB) for 5 min and wash with distilled water to stop color development; 11) After hematoxylin counterstaining, washing, and differentiation, fully washed and returned to blue; 12) Conventional dehydration and transparent, neutral gum sealing. Quantification of the IHC-positive signals of *FDX1* was performed by ImageJ and the IHC Profiler plugin.

### Cell culture and experiments

The 786-O cell line was obtained from the Central Laboratory of Tumor Hospital (Liaoning, China). RPMI 1640 containing 10% fetal bovine serum was used to culture the cell line at 37°C in a humidified atmosphere containing 5% CO_2_. Small interfering RNA (siRNA) was purchased from Sangon Biotech (Shanghai, China). The 786-O cells were transfected with 50 nM *FDX1* siRNAs using LIPO3000. Elesclomol (10 nM) and CuCl_2_ (10 μM) were used to induce cuproptosis. RT-PCR was used to evaluate the knockdown efficiency after 48 h of transfection. The following siRNA sequences were used: hFDX1-378-F, GUCCACUUUAUAAACCGUGAUTT; hFDX1-378-R, AUCACGGUUUAUAAAGUGGACTT. The primers were as follows: FDX1-F, TTCAACCTGTCACCTCATCTTTG; FDX1-R, TGCCAGATCGAGCATGTCATT; GAPDH-F, GGA GCGAGATCCCTCCAAAAT; and GAPDH-R, GGCTGTTGT CATACTTCTCATGG. The Cell Counting Kit-8 (CCK-8) was used to calculate the cell viability.

### Statistical analysis

Spearman's rank correlation coefficient was utilized to discover the strength of a link between two independent sets of data. The Wilcoxon rank-sum test was used to calculate the significance of the differences in methylation, transcription, protein, and phosphorylation levels between normal and tumor groups. All analyses were conducted in R version 3.6.3, and data visualization was accomplished by ggplot2. In this study, p < 0.05 is regarded as statistically significant (ns, not significant, p ≥ 0.05; *p < 0.05; **p < 0.01; ***p < 0.001).

## Results

### *FDX1* expression analysis at transcription, protein, phosphorylation, and promoter methylation levels

**Figure 1:**
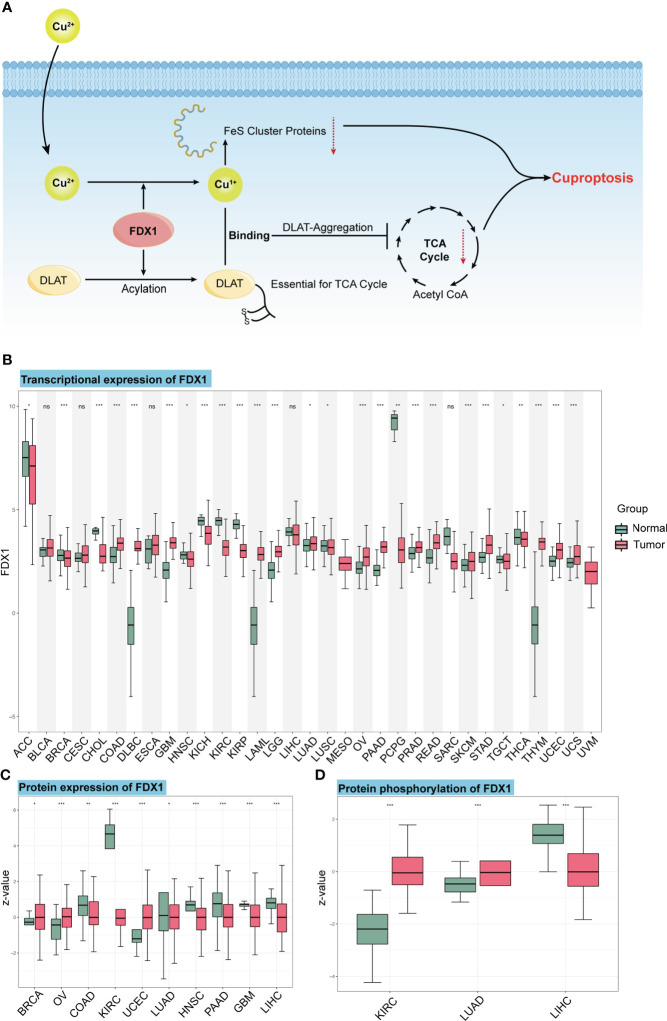
The mechanism of cuproptosis and expression of *FDX1* at different levels. **(A)** *FDX1* works as the core regulatory gene in the process of copper-induced death. **(B)** *FDX1* is differently expressed in the 33 tumors at the transcriptional level. **(C, D)** Protein level and phosphorylation of *FDX1* in different tumors and normal tissues based on data from CTPAC. ns, not significant, p ≥ 0.05; *p < 0.05; **p < 0.01; ***p < 0.001. (Cu, copper; *FDX1*, ferredoxin 1; TCA, tricarboxylic acid; DLAT, dihydrolipoamide S-acetyltransferase).

When Cu^2+^ was transported into the cell by its carrier, it could be reduced to Cu^1+^ by *FDX1*. Meanwhile, *FDX1* could regulate the acylation of dihydrolipoamide S-succinyl transferase (DLAT), an indispensable component of the TCA cycle-related pyruvate dehydrogenase complex. Increased Cu^1+^ caused cuproptosis by binding to DLAT and decreasing Fe-S cluster proteins (**Figure 1A**). In this study, we first analyzed the transcription level of *FDX1* in the 33 tumors (**Figure 1B**), and it was found that *FDX1* was highly expressed in 15 tumors: colon adenocarcinoma (COAD), diffuse large B-cell lymphoma (DLBC), glioblastoma multiforme (GBM), acute myeloid leukemia (LAML), brain lower-grade glioma (LGG), lung adenocarcinoma (LUAD), ovarian serous cystadenocarcinoma (OV), pancreatic adenocarcinoma (PAAD), prostate adenocarcinoma (PRAD), rectum adenocarcinoma (READ), skin cutaneous melanoma (SKCM), stomach adenocarcinoma (STAD), thymoma (THYM), uterine corpus endometrial carcinoma (UCEC), and uterine carcinosarcoma (UCS) (p < 0.05). *FDX1* levels in 11 other tumors were lower compared to normal tissues: adrenocortical carcinoma (ACC), breast invasive carcinoma (BRCA), cholangiocarcinoma (CHOL), head and neck squamous cell carcinoma (HNSC), kidney chromophobe (KICH), KIRC, kidney renal papillary cell carcinoma (KIRP), lung squamous cell carcinoma (LUSC), pheochromocytoma and paraganglioma (PCPG), testicular germ cell tumor (TGCT), and thyroid carcinoma (THCA) (p < 0.05). While in bladder urothelial carcinoma (BLCA), cervical squamous cell carcinoma and endocervical adenocarcinoma (CESC), esophageal carcinoma (ESCA), liver hepatocellular carcinoma (LIHC), and sarcoma (SARC), *FDX1* showed no significant expression difference compared to normal tissues.

We also used the CTPAC to analyze the protein level of *FDX1* in 10 tumors (**Figure 1C**). It was found that *FDX1* expression was higher in BRCA, OV, and UCEC compared to that in normal tissues (p < 0.05), while it was lower in COAD, KIRC, LUAD, HNSC, PAAD, GBM, and LIHC (p < 0.05).

As an efficient regulator of protein functions, the phosphorylation level of *FDX1* was also assessed based on CTPAC datasets (**Figure 1D**). The result represents the higher phosphorylation level of S177 in KIRC and LUAD and the lower phosphorylation level of S159 in LIHC.

**Figure 2:**
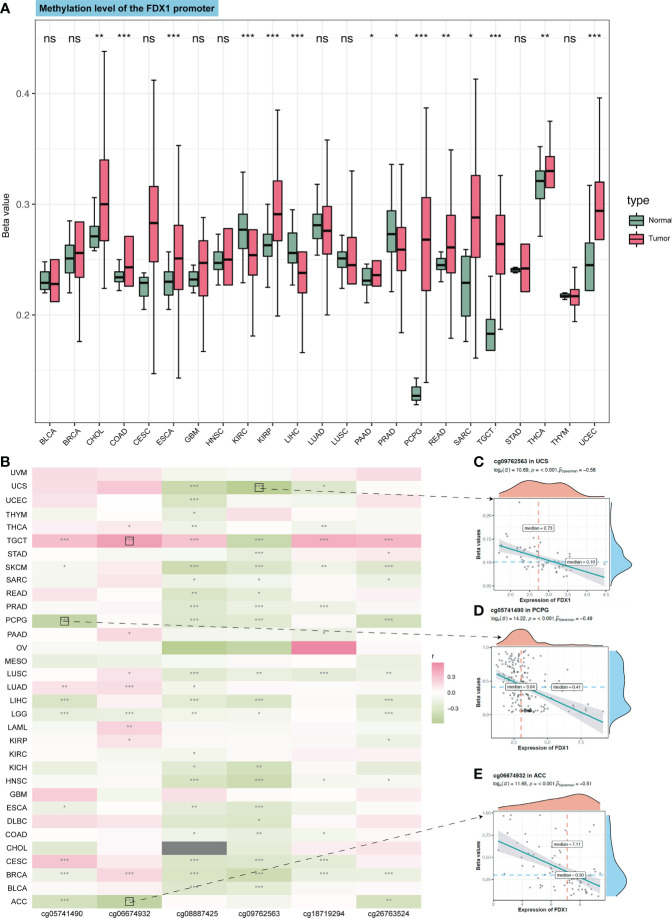
Methylation of the *FDX1* promoter and correlation between methylation probes and the *FDX1* expression. **(A)** Methylation beta value of the *FDX1* promoter in tumors and normal tissues. **(B)** Correlation between the *FDX1* expression and six *FDX1* promoter methylation probes. The correlation with the highest r value and p < 0.001 was marked with black boxes. **(C–E)** The spot correlation plot between the methylation of probes and the *FDX1* expression in UCS, PCPG, and ACC. ns, not significant, p ≥ 0.05; *p < 0.05; **p < 0.01; ***p < 0.001.

DNA methylation is the main epigenetic form of mammalian gene expression regulation. We got the methylation level of the *FDX1* promoter from CTPAC datasets (**Figure 2A**). It showed that CHOL, COAD, ESCA, KIRP, PAAD, PCPG, READ, SARC, TGCT, THCA, and USEC had a higher level of *FDX1* promoter methylation (p < 0.05), while *FDX1* in CHOL, KIRP, PCPG, TGCT, and THCA was lowly expressed. The *FDX1* promoter in KIRC, LIHC, and PRAD was lowly methylated (p < 0.05), but only PRAD had a higher expression of *FDX1*. We also analyzed the relation between six *FDX1* promoter methylation probes and the expression of *FDX1* (**Figures 2B–E**). As shown in the heatmap, four correlations with the top r value plus p-value <0.001 were framed by black boxes. The expression of *FDX1* was negatively correlated with the methylation probe in ACC and PCPG (**Figures 2D, E**), which may account for their lower transcription expression of *FDX1* compared to that in normal tissues.

### *FDX1* as a prognostic factor in different tumors

**Figure 3:**
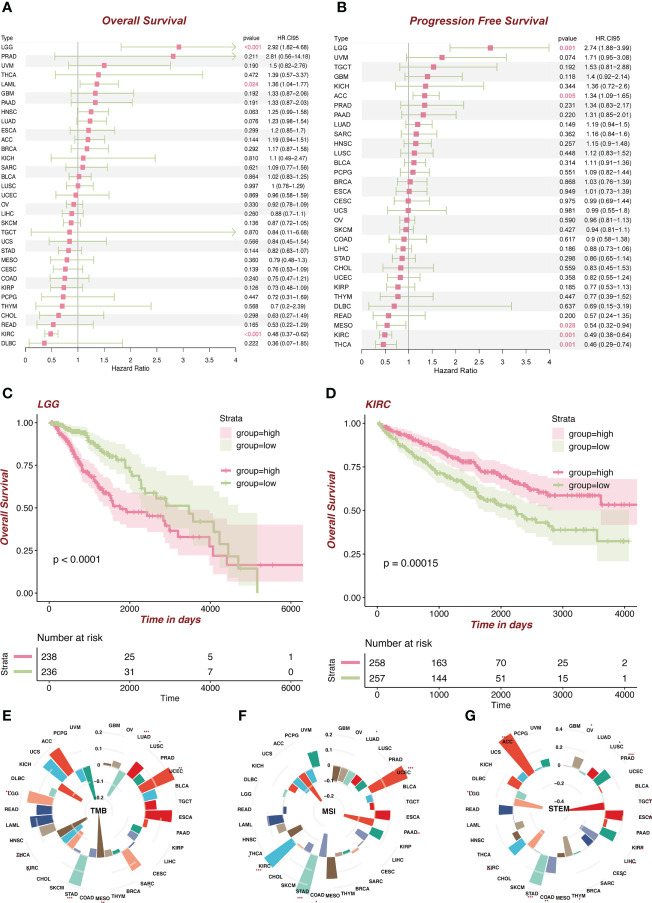
Survival analysis of *FDX1* in tumors and TMB, MSI, and stemness analysis of *FDX1*. **(A)** Overall survival in different tumors based on the *FDX1* expression 50% cutoff grouping. **(B)** Progression-free survival in different tumors based on the *FDX1* expression 50% cutoff grouping. **(C, D)** The Kaplan–Meier plot of LGG and KIRC based on the *FDX1* expression 50% cutoff grouping. **(E–G)** Tumor mutant burden (TMB), microsatellite instability (MSI), and stemness analysis of *FDX1.* ns, not significant, p ≥ 0.05; *p < 0.05; **p < 0.01; ***p < 0.001.

We divided the patients into the high (the top 50%) and low (the bottom 50%) *FDX1* expression group. Cox regression analysis (**Figure 3A**) showed that *FDX1* in LGG [hazard ratio (HR) = 2.92, p < 0.001, **Figure 3C**] and LAML (HR = 1.36, p = 0.024) was a risk factor of patients’ OS, while *FDX1* in KIRC (HR = 0.48, p < 0.001, **Figure 3D**, demographic information in **Supplementary Table S1**) was a protective factor. Regarding PFS (**Figure 3B**), a high expression of *FDX1* was associated with poor PFS in LGG (HR = 2.74, p = 0.001) and ACC (HR = 1.34, p = 0.005), while a high expression of *FDX1* could predict favorable PFS in mesothelioma (MESO) (HR = 0.54, p = 0.028), KIRC (HR = 0.49, p = 0.001), and THCA (HR = 0.46, p = 0.001). Furthermore, based on the optimal cutoff value instead of 50%, *FDX1* played a prognostic role in OS in ACC, COAD, LGG, HNSC, KIRC, LIHC, KIRP, and LAML (**Supplementary Figure S1**). In addition, *FDX1* could work as a prognostic factor of PFS in nine types of tumors (**Supplementary Figure S2**).

### Tumor mutant burden, microsatellite instability, and stemness analysis of *FDX1*

We subsequently analyzed the association between *FDX1* expression and TMB, MSI, and stemness. TMB represents levels of gene mutations in their tumor cells (19). We noted that *FDX1* expression was positively related to TMB in UCEC, SARC, HNSC, STAD, and LGG but negatively in THCA, KIRC, MESO, and LUAD (**Figure 3E**). MSI refers to the failure of the DNA mismatch repair (MMR) mechanism during DNA replication (20). *FDX1* expression was positively related to MSI in KIRC, STAD, and UCEC but negatively in LUAD, THCA, COAD, and PAAD (**Figure 3F**). Stemness refers to the ability of self-renewal and to differentiate into various cell types (21). High expression of *FDX1* was related to higher stemness, especially in ACC (**Figure 3G**).

### Immune infiltration and immune-related gene analysis

**Figure 4:**
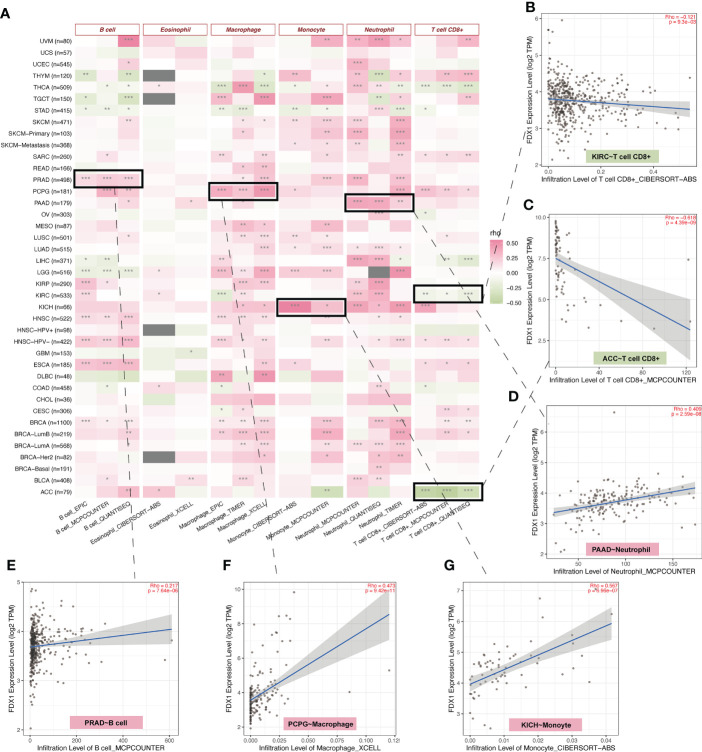
Immune infiltration analysis of *FDX1*. **(A)** The correlation heatmap between the six immune cells and *FDX1* expression in tumors based on two or three algorithms. **(B–G)** The correlation plot of those having consistent r values based on the different algorithms. ns, not significant, p ≥ 0.05; *p < 0.05; **p < 0.01; ***p < 0.001.

The TCA cycle has been highlighted in the immune system (22, 23). As *FDX1* was related to the TCA cycle (1), it may influence tumors’ immune infiltration and expression of immune-related genes. Therefore, we used TIMER2 to do the immune estimation based on several algorithms including EPIC, MCPcounter, quanTIseq, CIBERSORT-ABS, xCELL, and TIMER (**Figure 4A**). Correlations between *FDX1* and immune cells that were consistent among two or three algorithms were regarded as meaningful (**Figures 4B–G**). High infiltrated B cell was positively related to *FDX1* in PRAD, HNSC, ESCA, and BRCA and was negatively related to *FDX1* in LGG. Highly infiltrated macrophage was positively correlated with *FDX1* in PCPG, LGG, HNSC, and BRCA. Monocytes were positively associated with *FDX1* in SKCM, LUSC, LUAD, LGG, and KICH. As for neutrophils, the positive correlation could be found in UVM, PAAD, LUAD, KICH, and BRCA. CD8^+^ T cells obtained more negative correlations with *FDX1*, especially in KIRC, ACC, and THCA. There were no meaningful results regarding eosinophils. To learn more about the possible relationship between *FDX1* and immunity, we analyzed the correlation between *FDX1* and a bunch of classical immune inhibitory and stimulatory genes (15). It could be found that both the inhibitory and stimulatory genes were positively associated with *FDX1* in most tumors, including UVM, SKCM, SARC, READ, PRAD, PAAD, OV, and LGG. But in UCS, LUSC, MESO, LAML, DLBC, COAD, CHOL, and BLCA, there was no significant association. In THCA, there were high positive associations between *FDX1* and inhibitory genes such as *EDNRB* (r = 0.58, p < 0.001) or *VEGFA* (r = 0.63, p < 0.001). However, in ACC, most of the inhibitory and stimulatory genes were negatively related to *FDX1* (**Supplementary Figure S3**).

### Single-cell analysis of *FDX1*

**Figure 5:**
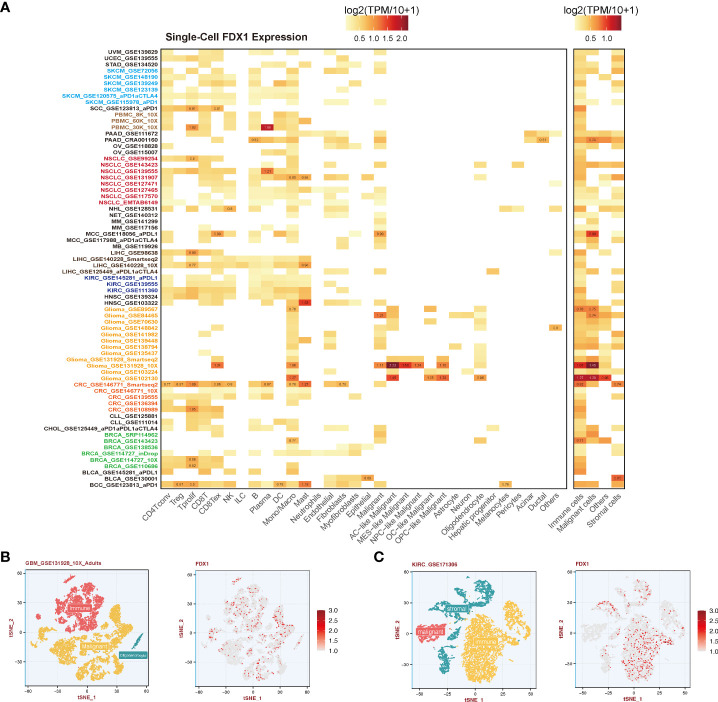
Single-cell analysis of *FDX1* expression. **(A)** Left: *FDX1* expression in different cells. Right: *FDX1* expression based on malignancy classification. **(B, C)** Single-cell profile and *FDX1* expression in GBM and KIRC.

To further understand the relationship between the tumor environment and *FDX1*, we used current public single-cell RNA sequence data to depict the expression portrait of *FDX1* across all available tumors (**Figure 5A**). According to the malignancy cell type annotation, immune cells in most tumors took the main responsibility for *FDX1* expression if we divided cells into immune cells, tumor cells, stromal cells, and other cells. Malignant cells in BRCA, GBM, MCC, PAAD, and SKCM obtained a stronger *FDX1* expression ability than other tumor types. As for stromal cells, BLCA and CRC maintained a high *FDX1* expression in stromal cells.

To be specific, immune cells including CD4 T cells, CD8 T cells, regulatory T cells (Tregs), natural killer (NK) cells, and dendritic cells (DCs) expressed *FDX1* in most tumors except for GBM. *FDX1* could be detected in almost all macrophages and monocytes in different tumors. In GBM, it was found that astrocyte (AC) - like malignant cells were the highest *FDX1*-expressing cells. The visualization of single-cell FDX1 expression in GBM and KIRC could be seen in **Figures  5B, C**.

### Correlation analysis between *FDX1* and CD274 (PD-L1) and the role of *FDX1* in immunotherapy

**Figure 6:**
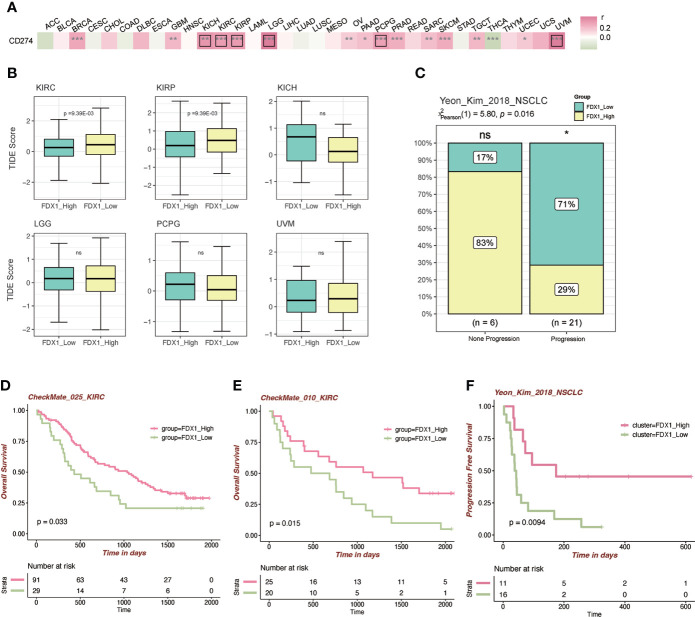
Immunotherapy-related analysis of *FDX1.* **(A)** Correlation between *FDX1* and CD274 (PD-L1) in the TCGA tumor cohorts. The black box means r > 0.3 and p < 0.05. **(B)** The TIDE scores between the high- and low-*FDX1* groups in the tumors. **(C)** The distribution between immunotherapy progression and *FDX1* expression. **(D–F)** The Kaplan–Meier plot of patients under immunotherapy between the high and low *FDX1* expression groups. ns p ≥ 0.05, *p < 0.05, **p< 0.01, ***p < 0.001.

To further figure out the relationship between *FDX1* and immunotherapy, the correlation analysis between *FDX1* and CD274 was done. It indicated that *FDX1* was positively related to CD274 in BRCA, GBM, KICH, KIRC, KIRP, LGG, OV, PAAD, PCPG, PRAD, SARC, SKCM, TGCT, UCEC, and UVM, especially in KICH, KIRC, KIRP, LGG, PCPG, and UVM (r > 0.3, p < 0.05, **Figure 6A**). The TIDE algorithm was used to predict the ICB response (**Figure 6B**). It was found that the higher *FDX1* expression group in KIRC and KIRP had a lower TIDE score, predicting a favorable response to ICB than the low-*FDX1* group. Then, two PD-1 blockade cohorts of KIRC (CheckMate_025 and CheckMate_010) were included to illustrate that the high-*FDX1* group had a higher OS rate after the PD-1 blockade treatment (**Figures 6D, E**). Similar findings were observed in another NSCLC cohort and its corresponding methylation cohort with the treatment of anti-PD-1/PD-L1 (**Figures 6C, F** and **Supplementary Figure S4A**). Other cohorts such as SKCM patients under CTLA-4 blockade and ACT were selected, and the results showed that the high-*FDX1* group was associated with a better prognosis (**Supplementary Figures S4B, C**).

### *FDX1* expression analysis in KIRC by tissue microarray immunohistochemistry and silence of *FDX1* downregulated cuproptosis in KIRC

*FDX1* was lowly expressed in KIRC compared to normal tissues in both transcription and protein levels based on public datasets, and *FDX1* was a protective factor in both OS and PFS. Disease-specific survival analysis (KIRC as the main cause of death) showed that the high-*FDX1* group had a more favorable prognosis (**Supplementary Figure S5**). Multi-Cox regression also indicated the survival prognostic value of *FDX1* in KIRC (HR = 0.451, 95% CI = 0.312–0.653, p < 0.001, **Supplementary Figure S6**). Moreover, it was verified that higher *FDX1* was positively related to a more favorable KIRC overall prognosis in other cohort data such as E-MATB and GSE14378 (**Supplementary Figure S7**). Furthermore, the TIDE score and established cohorts suggest *FDX1* as an important potential predictive gene in KIRC. Thus, KIRC was selected to do further verification by IHC of KIRC tissue microarray and *in vitro* experiments.

**Figure 7:**
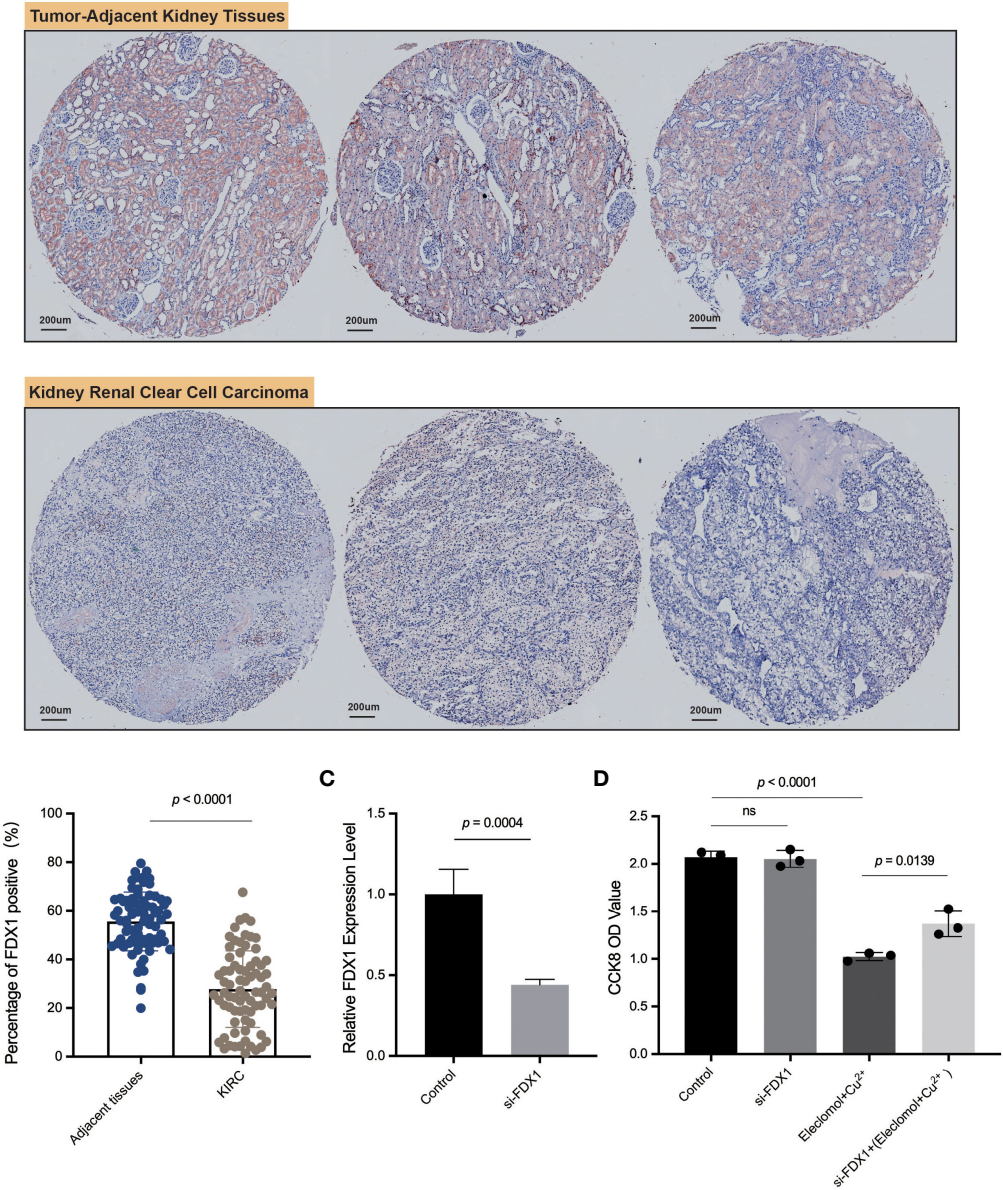
IHC of *FDX1* in the kidney renal clear cell carcinoma tissue microarray and *in vitro* experiments. **(A)** The pictures of IHC under the microscope. The *FDX1*-positive areas are tan-colored. **(B)** Comparison of the *FDX1*-positive IHC areas between tumor and adjacent normal tissues based on ImageJ quantification. **(C)** The relative expression of *FDX1* in 786-O cells transfected with si-*FDX1*. **(D)** The cell growth OD value (450 nm) assessed by CCK-8 kit after 24 h with the addition of elesclomol+Cu^2+^. A higher OD value means more living cells. The elesclomol+Cu^2+^ was used to induce cuproptosis. ns means not significant, p ≥ 0.05.

We applied ImageJ to quantify the expression of FDX1 and found that the *FDX1* level was higher in adjacent kidney tissues than that in KIRC tumor tissues (**Figures 7A, B**), consistent with our previous series analysis. As shown in **Figure 7A**, FDX1 was abundantly expressed in renal tubular cells, especially in normal tissues. This experiment further validated the differential expression level of FDX1 and suggested its favorable role in the treatment of KIRC.

To further explore the function of *FDX1* in KIRC, 786-O cells, one type of kidney renal clear tumor cell line, were transfected with siRNA against *FDX1* (**Figure 7C**). Downregulation of *FDX1* did not significantly affect 786-O cell proliferation, but the lack of *FDX1* could reverse elesclomol-copper–induced cell death (**Figure 7D**). These observations illustrated that a high *FDX1* expression may promote cuproptosis *in vitro*, which may be associated with a better prognosis and therapeutic efficacy.

### Gene set enrichment analysis of *FDX1* and the role of the NOTCH pathway

**Figure 8:**
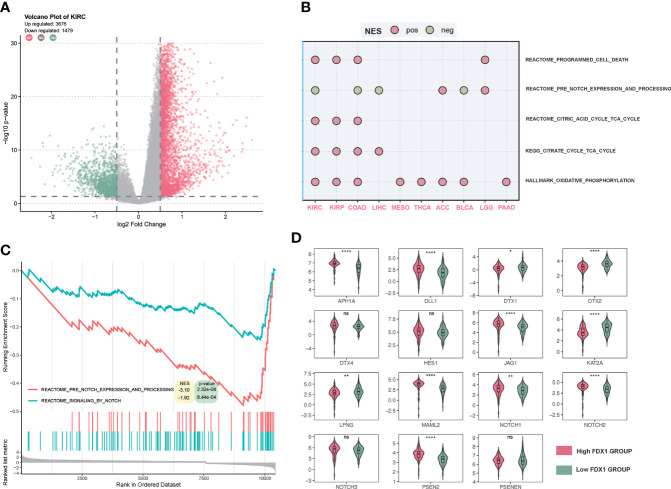
Gene set enrichment analysis (GSEA) of *FDX1* in KIRC. **(A)** Differentially expressed genes based on the *FDX1* expression 50% cutoff grouping and the volcano plot. The red spots mean upregulated genes, and the green spots mean downregulated genes. **(B)** Dot plot of GSEA in 10 cancer types. The red dot means that the gene set is enriched in the high-*FDX1* group, while the green dot means that the gene set is enriched in the low-*FDX1* group. **(C)** The GSEA plot of the NOTCH-related gene sets. **(D)** The expression of the 15 NOTCH pathway genes between high- and low-*FDX1* groups (50% cutoff). p ≥ 0.05, *p < 0.05, **p < 0.01, ****p < 0.0001.

To further figure out the function of *FDX1* in tumors, a GSEA of several cancer types was performed. As shown in **Figure 8A**, there were more than 4,000 differential genes between the high and low *FDX1* expression groups. We did a GSEA in several cancer types, and the enrichment pathways were presented such as TCA_CYCLE and OXIDATIVE_PHOSPHORYLATION, in which the TCA_CYCLE may be related to cuproptosis. Interestingly, the NOTCH pathway showed up in half of the cancer types, and NOTCH was negatively related to the *FDX1*-associated gene expression signature in KIRC, COAD, LIHC, and BLCA (**Figure 8B**). The GSEA plot of *FDX1* for KIRC was shown in **Figure 8C**, and it revealed the relationship between *FDX1* and the NOTCH pathway. We further compared the expression of 15 NOTCH pathway genes [intersection of HALLMARK_NOTCH_SIGNALING (32 genes) and KEGG_NOTCH_SIGNALING_PATHWAY (47 genes)] between the high- and low-*FDX1* groups. It appeared that the high-*FDX1* KIRC group had higher APH1A, DLL1, MAML2, PSEN2, JAG1, NOTCH1, and NOTCH2 and lower LFNG, DTX1, and DTX2 (**Figure 8D**, p < 0.05). Additional results indicated that FATTY ACID METABOLISM, UBIQUITIN-MEDIATED PROTEOLYSIS, OXIDATIVE PHOSPHORYLATION, MTOR SIGNALING, PROTEIN SECRETION, TG_BETA_SIGNALING, and PROCESSING_OF_SMAD1 may be positively related to the *FDX1*-associated gene expression signature (**Supplementary Figure S8**).

## Discussion

Cuproptosis could be induced by elesclomol, a kind of copper ionophore that transfers copper into cells (24). The overload of copper could promote the aggregation of lipoylated proteins in the TCA cycle and destabilization of Fe-S cluster proteins, finally resulting in this manner of cell death (1). Like ferroptosis, cuproptosis holds promise as an effective approach for tumor diseases. It is found that elesclomol could impair glioblastoma stem-like cell survival and tumor growth (25). Indeed, elesclomol is already under clinical trial in patients with stage IV melanoma. Researchers demonstrated that the addition of elesclomol to the traditional paclitaxel chemotherapy reveals a significantly improved median PFS in the low-lactate dehydrogenase (LDH) melanoma group (26). Low LDH indicated more reliance on mitochondrial respiration and the TCA cycle, which would be more sensitive to cuproptosis (1). Based on the result of another multicenter phase 2 trial, there was no significant improvement in response to paclitaxel plus elesclomol in patients with platinum-resistant recurrent ovarian carcinoma (27). As discussed above, the cuproptosis inducer, elesclomol, has been widely used in various tumors, but the real contribution of the key regulatory gene, *FDX1* remains unknown, and novel therapeutic strategies are urgently needed. As the key regulatory gene in cuproptosis, *FDX1* may serve as a target for novel agents and treatments. Given that *FDX1* was hardly reported in tumor therapy, we present here a comprehensive pan-cancer analysis of *FDX1*.

In the 33 tumors, *FDX1* was differently expressed in 28 types of tumors. This founding was also reported in other pan-cancer studies (28, 29). DNA methylation was commonly detectable in those tumors as seen in the heatmap of correlation between *FDX1* expression and *FDX1* promoter methylation probes. Higher *FDX1* promoter methylation in the tumor group might account for the low *FDX1* expression especially in CHOL, KIRP, PCPG, SARC, TGCT, and THCA. We also observed the opposite direction of methylation and expression changes in some tumors, which may be due to cellular heterogeneity. In the analysis of methylation sequencing data, traditional methods usually calculate the methylation values of all cytosine-guanine (CpG) dinucleotides sites in the promoter region, and then the average value was taken to represent the degree of methylation in the region. This approach ignores the cellular heterogeneity in the cells or tissue samples used for gene sequencing, that is, the methylation status of a locus may vary at the single-cell level (30). Moreover, the expression of *FDX1* mRNA was in accordance with the protein level in OV, KIRC, UCEC, and HNSC, which was not consistent in other tumors, suggesting that posttranscriptional modifications of *FDX1* expression may also exist.

In the survival analysis, at the 50% cutoff of *FDX1*, there was a statistically significant difference in OS for LGG, LAML, and KIRC tumor types and in PFS for LGG, ACC, MESO, KIRC, and THCA. We also carried out an analysis based on optimal cutoff values. The result showed a significant OS in ACC, COAD, HNSC, LIHC, and KIRP with a significant PFS in BLCA, PAAD, LIHC, and UVM, which means that a higher threshold of *FDX1* expression difference may be required in these tumors to optimize outcomes.

Cancer stem cells refer to the tumor cells that contribute to the maintenance and long-term growth of tumors (31). The presence of cancer stem cells in cancer tissue boosts tumor growth, progression, and metastasis (32). The stemness analysis indicated that a high *FDX1* expression in ACC, COAD, and KIRP was correlated with high cancer stemness, which may account for the differences in the prognostic value among various tumor types.

Immuno-oncology has revolutionized cancer treatment (33). Tumorigenesis and progression are usually accompanied by the recruitment of numerous immune cells (34). There were barely any studies about *FDX1* and immune cells. Interestingly, our study showed that *FDX1* had a long-standing relationship with immune cells. Based on immune infiltration, we found that *FDX1* expression was highly infiltrated with macrophages in most tumors. The single-cell *FDX1* expression analysis also revealed that *FDX1* is ubiquitously expressed in immune cells, especially with a high level of expression in monocytes and macrophages. It was reported that the copper transporter ATP7A expression in THP-1 cells (a monocyte/macrophage model cell line) played a role in controlling intracellular copper levels and the oxidation of lipoproteins (35). Whether this process would orchestrate the expression of *FDX1* needs further analysis and experiments.

Regarding TMB and MSI, the two indexes indicated the possibility of benefit from ICB treatment (19, 36). In our research, *FDX1* expression was related to TMB in 10 tumors and MSI in seven tumors, which suggests that *FDX1* could affect patients’ response to immune checkpoint therapy. However, such a relationship was not observed in most tumors; we speculate that the effect would be limited. Overall, the precise function of *FDX1* in the immune cells or system needs further analysis and investigation.

To further analyze the role of *FDX1* in immunotherapy, the correlation analysis between *FDX1* and CD274 (PD-L1) was conducted. It revealed that *FDX1* was positively related to CD274 in most tumors. TIDE scores showed that higher *FDX1* in KIRC would have a better response to immunotherapy. Established cohorts of KIRC verified the prediction as shown in **Figure 6**. A similar situation was observed in NSCLC patients. The response rate of PD-1/PD-L1 inhibitors is unsatisfactory in a majority of tumor patients (37). Thus, finding biomarkers that could effectively predict the efficacy of PD-1/PD-L1 inhibitors is meaningful for immunotherapy. These data imply that *FDX1* may have attractive potential as a biomarker for tumor immunotherapy.

Among the 33 tumors, *FDX1* was downregulated in the KIRC group than normal tissues at the transcription and protein levels. The analysis revealed that the phosphorylation level of *FDX1* was upregulated in KIRC. Several studies demonstrated that phosphorylation of *FDX1* could inhibit its function, which may facilitate tumor growth (38, 39). The survival analysis found that *FDX1* played a significant protective role in the OS and PFS of KIRC patients. The immunotherapy analysis showed the potential role of *FDX1* in KIRC. All of these results indicated that KIRC was the tumor with the greatest potential to apply *FDX1*-related cuproptosis for therapy. Consistently, our tissue microarray analysis verified the lower expression level of *FDX1* in KIRC than that in normal tissues. Our *in vitro* experiments found that the silence of *FDX1* could downregulate elesclomol-copper–induced cell death (cuproptosis). Another study also proved the lower expression level of *FDX1* in KIRC (40). This finding revealed that kidney renal clear tumor cells with a high *FDX1* expression were sensitive to cuproptosis, which may account for a better survival prognosis in patients with a high *FDX1* level.

In the GSEA, it was interesting that the NOTCH-related pathway was negatively related to *FDX1* in several cancer types, which might be the common features of *FDX1*-related genes. To further figure out the role of *FDX1* in KIRC, our GSEA suggested that *FDX1* was mainly enriched in the TCA cycle, ubiquitin-mediated proteolysis, and the NOTCH pathway in KIRC. The TCA cycle gene set enrichment indicated a tight association with cuproptosis, which is consistent with the mechanisms elucidated previously. Ubiquitin was reported before that copper complexes as ubiquitin-proteasome system (UPS) inhibitors could enhance the effect of cancer treatment compared to UPS itself (41). There are no studies regarding the connection between *FDX1* and NOTCH. However, the NOTCH played an important role in KIRC. It was found that alternation of NOTCH could predict better prognosis in KIRC patients (42) and that the NOTCH gene-based signature had prognostic values (43). The downregulation of NOTCH target genes DTX1 and DTX2 in the high-*FDX1* group might be the connection between *FDX1* and NOTCH. The NOTCH might be a bridge between *FDX1* and its prognostic role in immunotherapy. Several studies indicated the potential crosstalk between the NOTCH and the PD-L1/PD-1 axis (44, 45). However, the underlying regulation of *FDX1* is still unclear. Further experiments are needed to figure out the mechanism.

There are several limitations in this study. Firstly, the function of *FDX1* under cuproptosis inducers was only verified in KIRC cells; more investigations involving other tumor types are essential. Secondly, although *FDX1* was the core regulator in cuproptosis, other cuproptosis-related genes may be involved in the process of tumorigenesis, which is needed to be discussed in future analyses. Thirdly, data on survival benefits from multicenter studies are still lacking.

## Conclusions

Cuproptosis opens a new area of investigation regarding programmed cellular death. Not only broader therapeutic applications but also novel agents for tumor treatment are deserving of ongoing exploration. The functions of *FDX1* in tumorigenesis, as the core gene in the regulation of cuproptosis, remain unclear. In this study, we provide a comprehensive pan-cancer analysis of *FDX1* and have shown that the distinct expression of *FDX1* correlates with the survival outcome of patients with diverse cancers. According to the immune infiltration, single-cell analysis, and immunotherapy analysis, *FDX1* may also participate in tumor immunity regulation, nevertheless, by an unidentified mechanism. KIRC was sensitive to cuproptosis, and *FDX1* played an important role in the process. The underlying connection between *FDX1* and the NOTCH pathway in KIRC needs further exploration. Collectively, the current results begin to help us understand the crucial role of *FDX1* in tumor biology and provide a novel direction for the application of cuproptosis in future tumor target therapies.

## Data availability statement

The datasets presented in this study can be found in online repositories. The names of the repository/repositories and accession number(s) can be found in the article/**Supplementary Material**.

## Author contributions

JX wrote the manuscript. ZH finished the whole analysis. QC and JL designed the study and revised the manuscript. HC, HZ, PL, JZ and XW conducted the microarray experiments and helped the writing of the manuscript. All authors contributed to the article and approved the submitted version.
