# Supplementary material for: Multi-omics pan-cancer study of cuproptosis core gene FDX1 and its role in kidney renal clear cell carcinoma
Source: Front Immunol. 2022 Dec 20;13:981764. doi: 10.3389/fimmu.2022.981764 (PMC9810262; doi:10.3389/fimmu.2022.981764)
Supplement: Supplementary file 1 [file DataSheet_1.pdf]

Supplement Table 1 The demographic information of KIRC patients.

Characteristic	Low expression of FDX1	High expression of FDX1	p
n	269	270	
T stage, n (%)			0.019
T1	121 (22.4%)	157 (29.1%)	
T2	38 (7.1%)	33 (6.1%)	
T3	103 (19.1%)	76 (14.1%)	
T4	7 (1.3%)	4 (0.7%)	
N stage, n (%)			0.191
N0	117 (45.5%)	124 (48.2%)	
N1	11 (4.3%)	5 (1.9%)	
M stage, n (%)			0.090
M0	210 (41.5%)	218 (43.1%)	
M1	47 (9.3%)	31 (6.1%)	
Age, median (IQR)	61 (53, 72)	60 (51, 69)	0.273

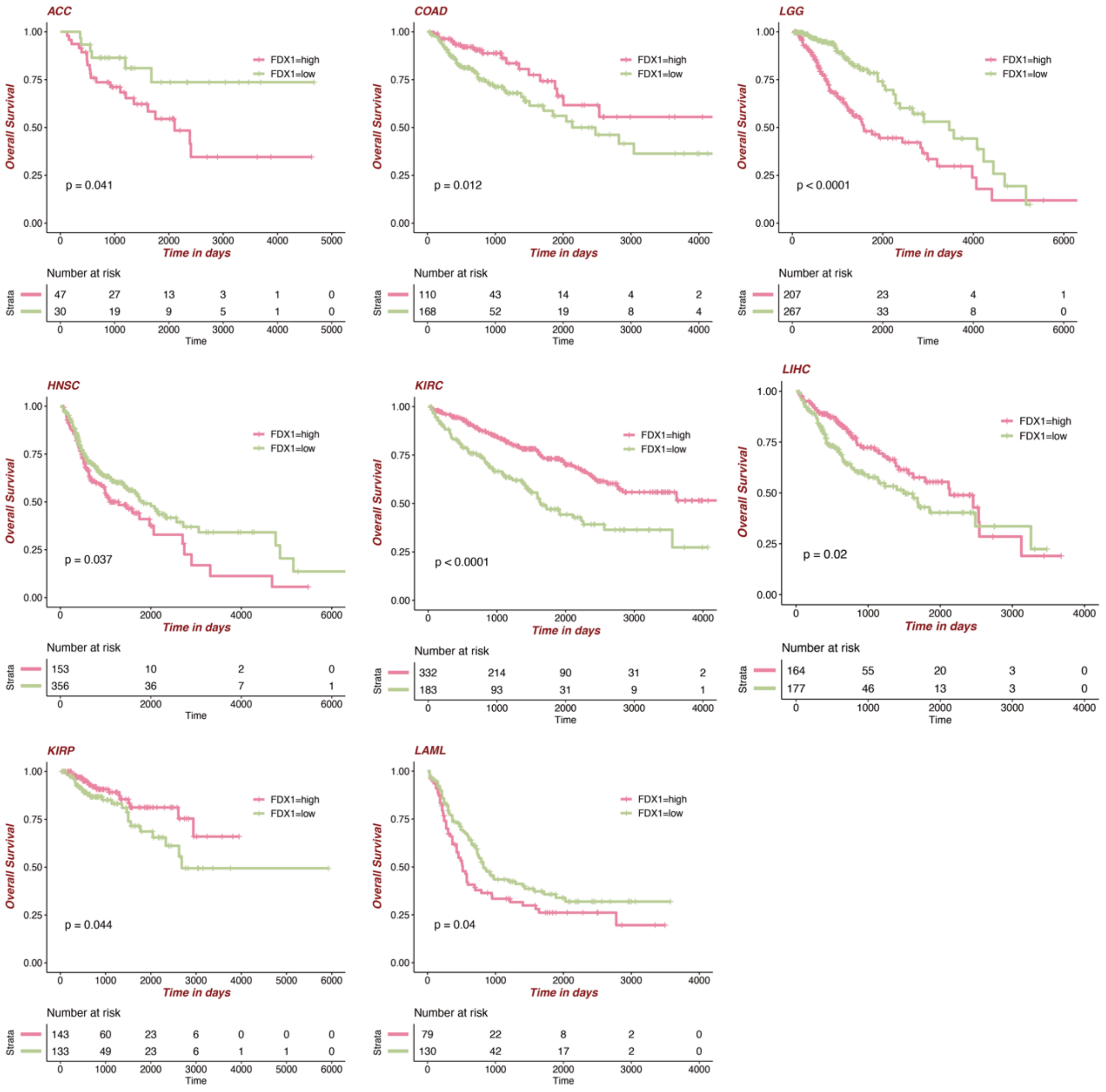

Supplement Figure 1 The Kaplan-Meier plot of overall survival in different tumors based on the optimal survival cut-off value of FDX1 expression.

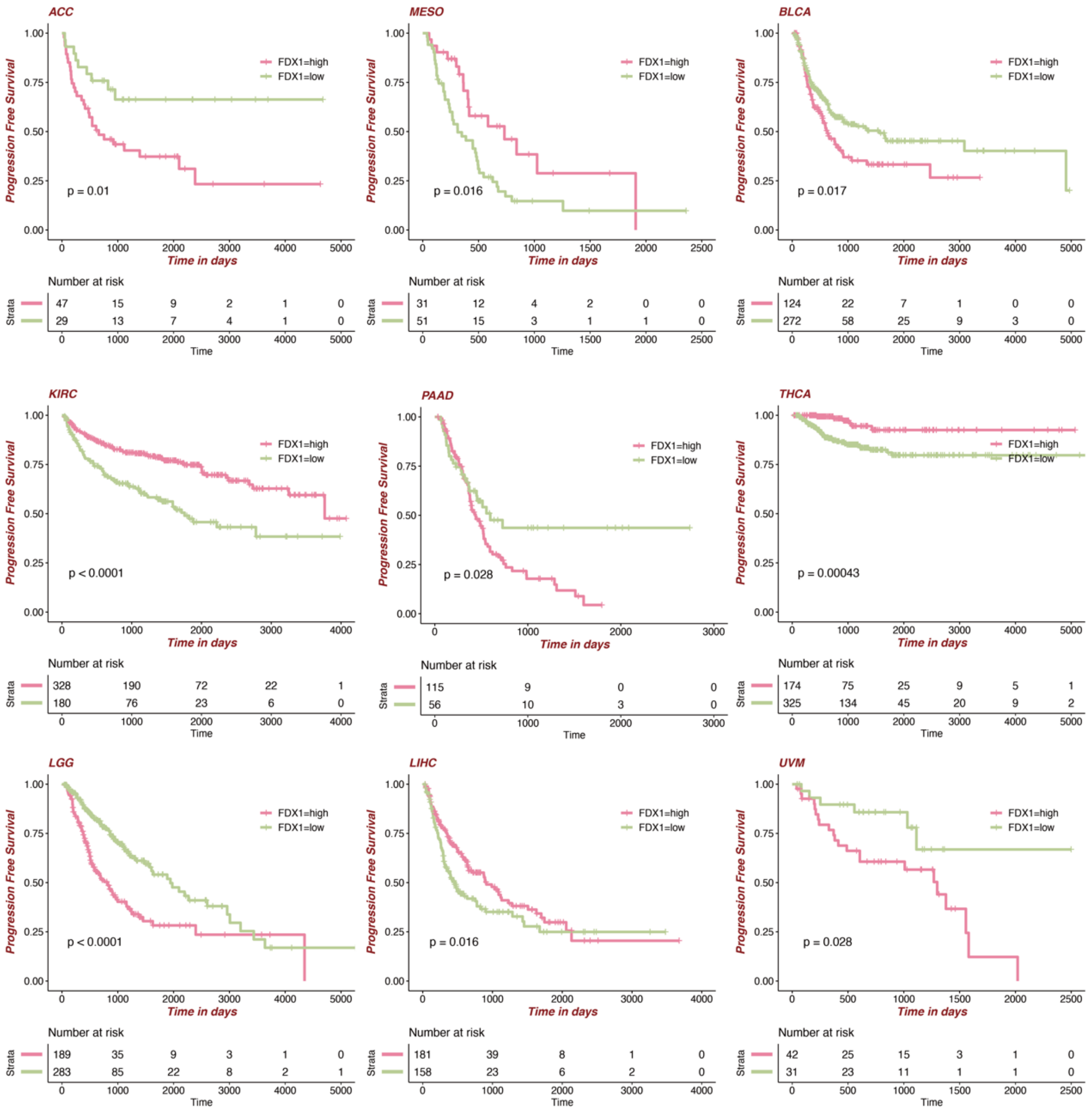

Supplement Figure 2 The Kaplan-Meier plot of progression-free survival in different tumors based on optimal survival cut-off value of FDX1 expression.

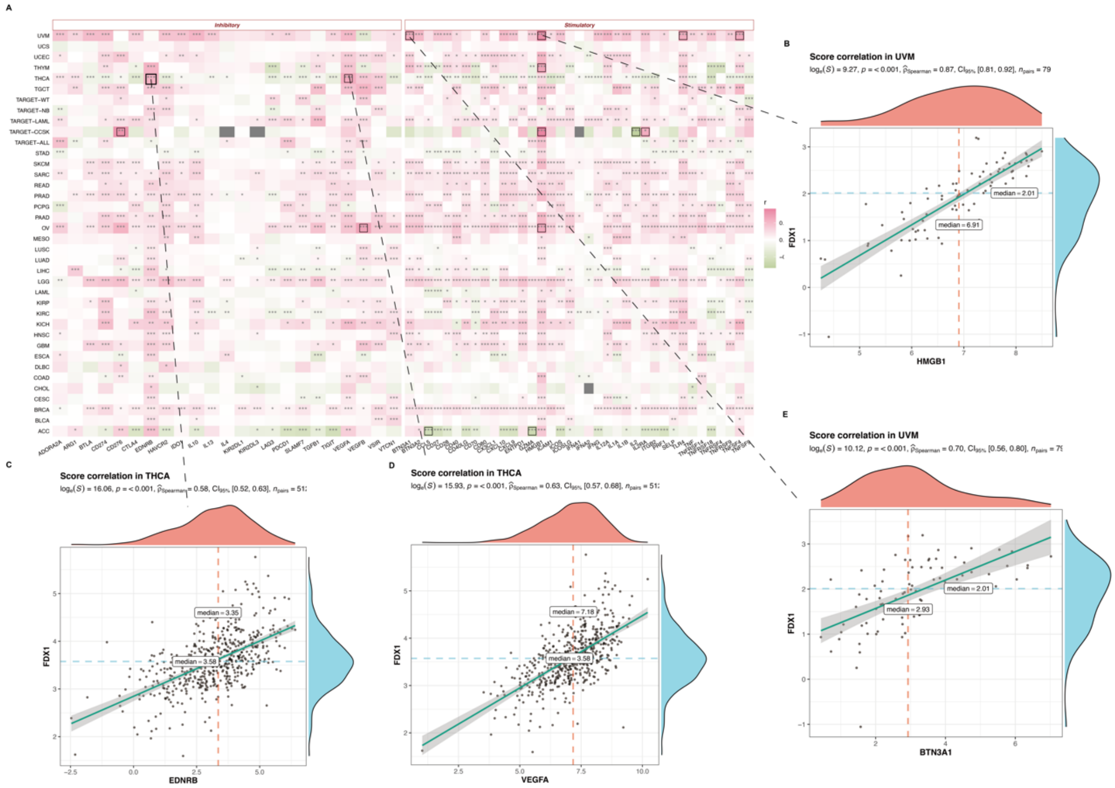

Supplement Figure 3 Correlation between immune genes and FDX1 expression.

(A) Correlation heatmap between inhibitory or stimulatory genes and FDX1. (B-E) The correlation scatter plot of those with correlation r value > 0.5. Those black boxes mean correlation r value > 0.5 and p<0.05.
p>=0.05, *p<0.05, **p<0.01, ***p<0.001

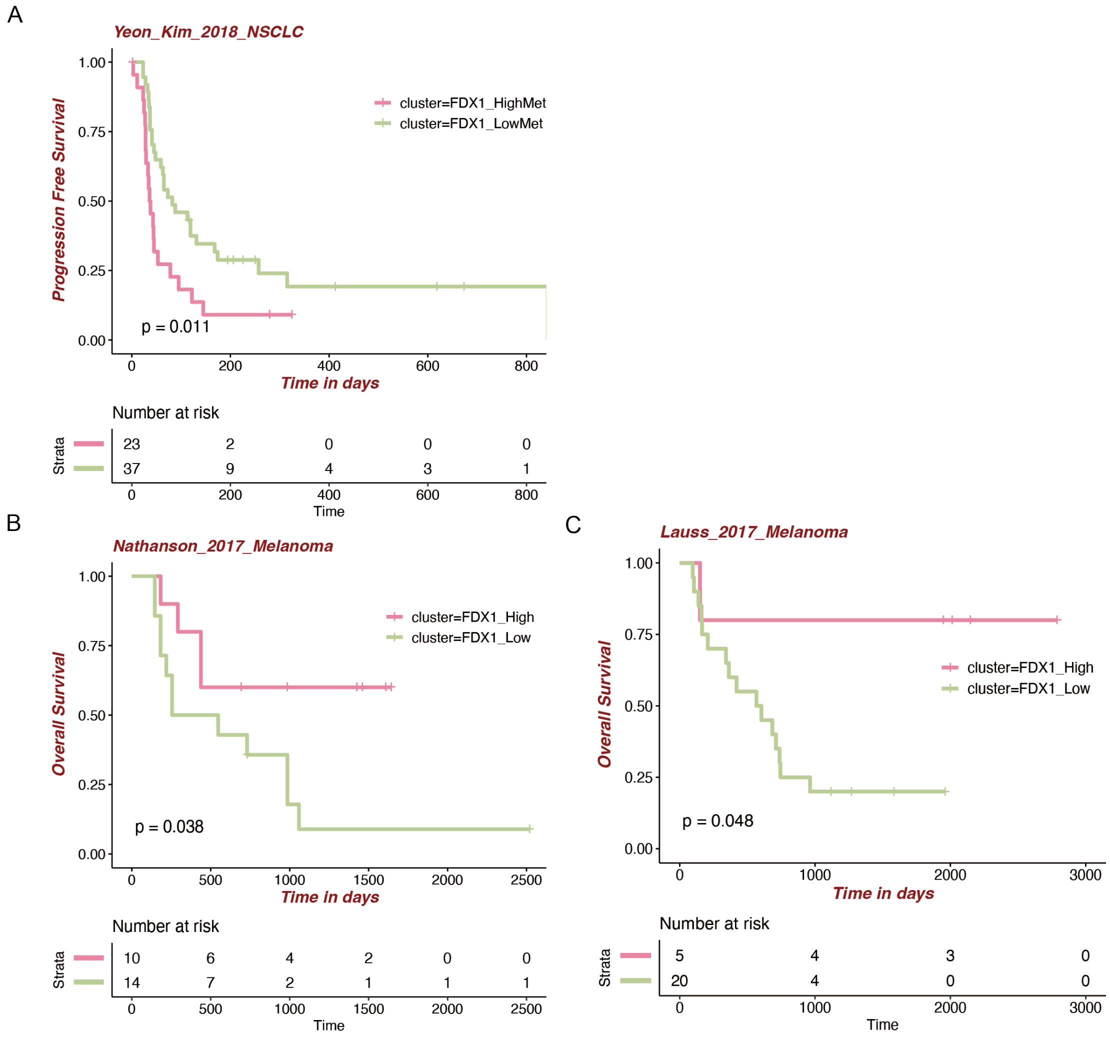

Supplement Figure 4 The Kaplan-Meier plot of patients' overall survival under immunotherapy between high and low FDX1 expression groups.

（A） The FDX1 methylation cohort of non-small cell lung cancer (NSCLC) with the treatment of anti-PD-1/PD-L1. A higher methylation level means a lower expression of FDX1.

（B） Nathanson_2017 cohort with melanoma patients treated with CTLA-4 blockade.

（C） Lauss_2017 cohort with melanoma patients under adoptive T-cell therapy

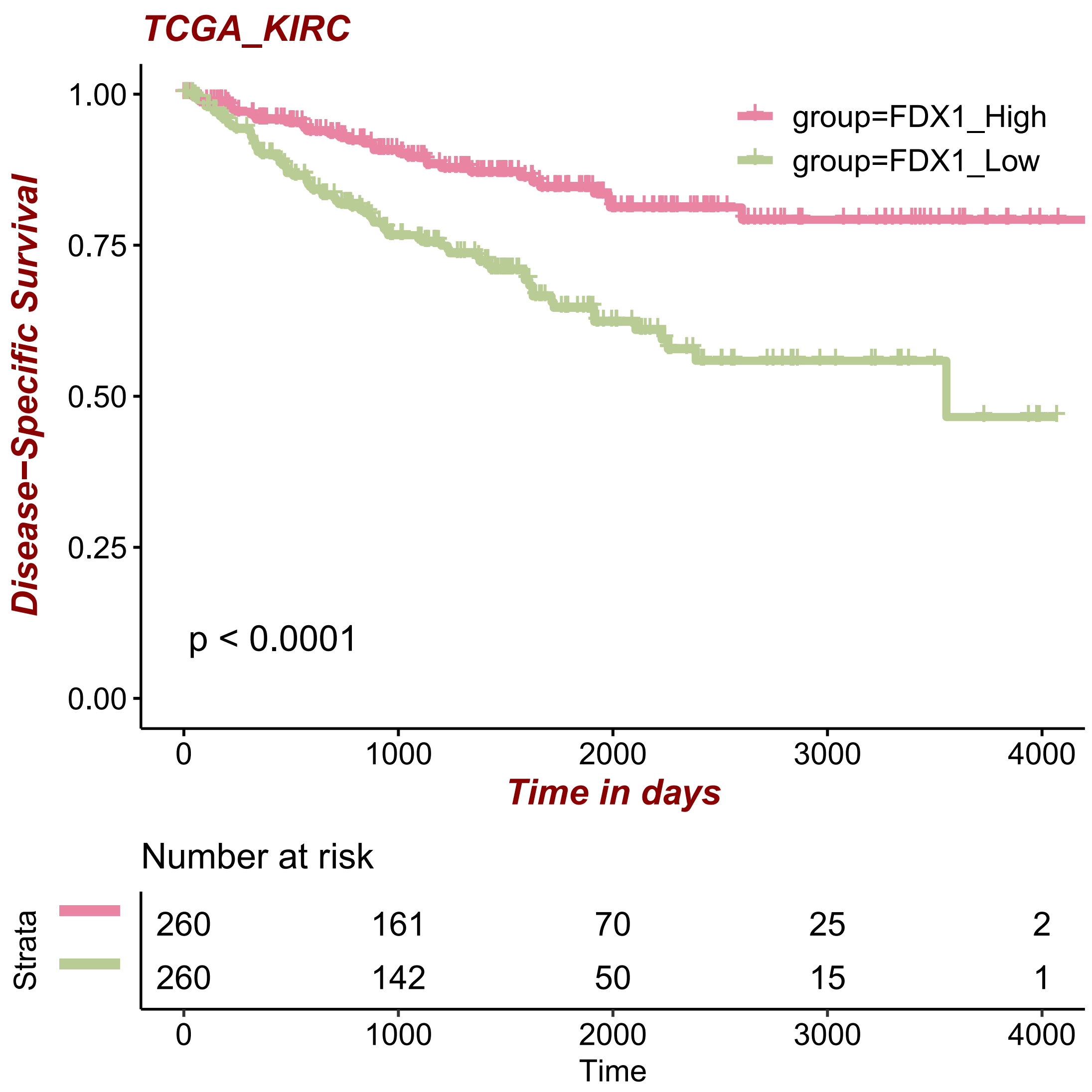

Supplement Figure 5 The Kaplan-Meier plot of disease-specific survival in KIRC.

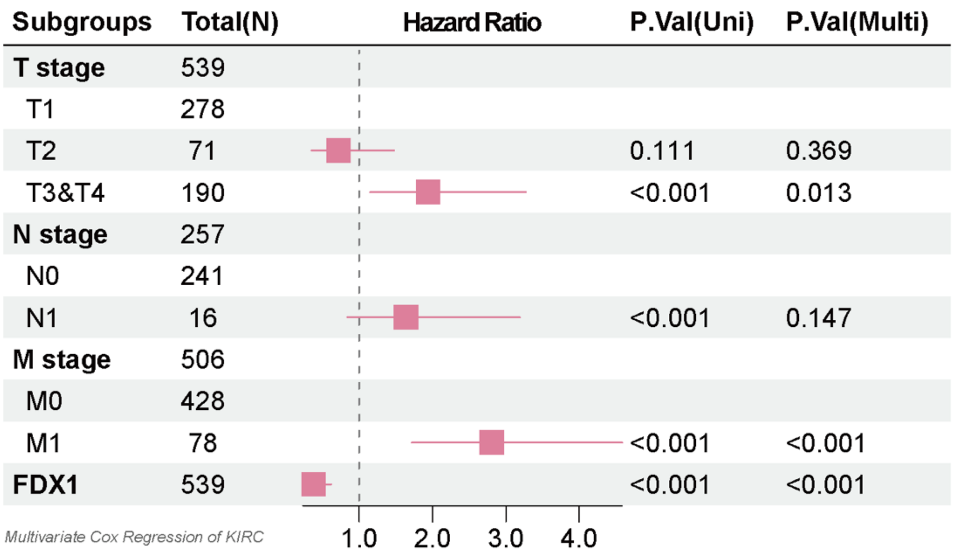

Supplement Figure 6 The Multi-Cox Regression in KIRC.

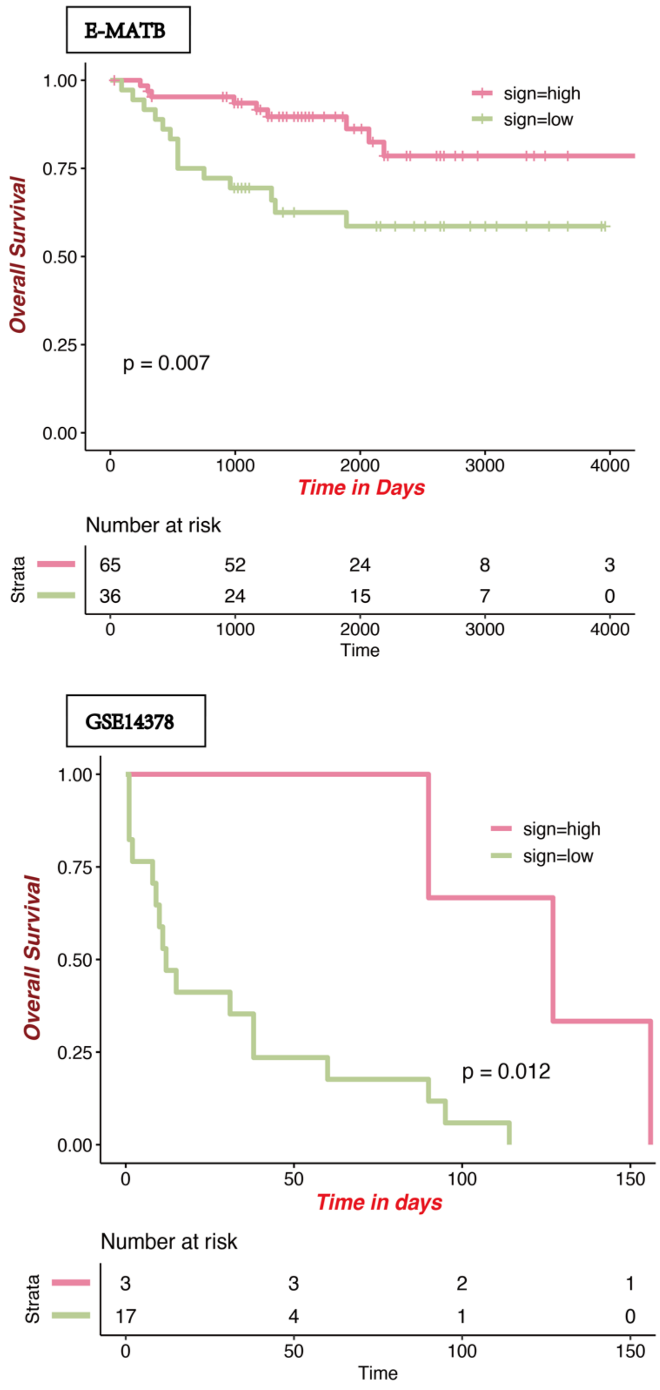

Supplement Figure 7 The Kaplan-Meier plot of overall survival in E-MTAB and GSE14378

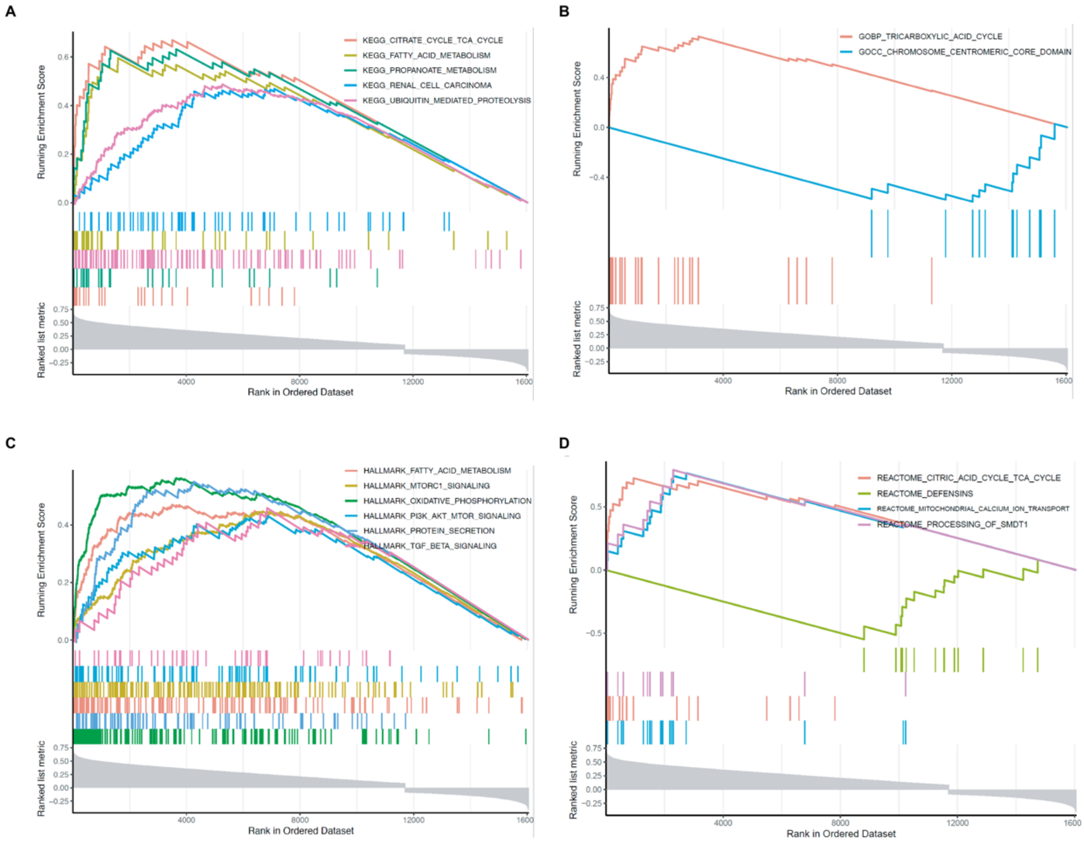

Supplement Figure 8 Detailed GSEA result of FDX1 in KIRC. (A) KEGG. (B) GO. (C) HALLMARK. (D) REACTOME.
